# Whispering-Gallery Mode Lasing in a Floating GaN Microdisk with a Vertical Slit

**DOI:** 10.1038/s41598-019-57118-y

**Published:** 2020-01-14

**Authors:** Gangyi Zhu, Jiaping Li, Nan Zhang, Xin Li, Jun Dai, Qiannan Cui, Qinghai Song, Chunxiang Xu, Yongjin Wang

**Affiliations:** 1 Peter Grünberg Research Centre, College of Telecommunications and Information Engineering, Nanjing University of Posts and Telecommunications, Nanjing, 210003 China; 2 State Key Laboratory of Bioelectronics, Southeast University, Nanjing, 210096 China; 3 State Key Laboratory on Tunable Laser Technology, Harbin Institute of Technology, Shenzhen, 518055 China; 4 College of Science, Jiangsu University of Science and Technology, Zhenjiang City, 212003 China

**Keywords:** Micro-optics, Microresonators

## Abstract

Controlling the lasing mode, emission direction, threshold, and quality factor of whispering-gallery mode lasing is important for practical applications such as optical interconnections, on-chip communications, trace detection, high-density storage, etc. In order to simultaneously control the mode and emission direction and to achieve a high-quality factor in a low-threshold whisper-gallery mode laser, such as a GaN floating microdisk, a novel fabrication design of a microdisk with a vertical slit is proposed. To demonstrate proof of concept, we experimentally measure whispering-gallery mode lasing spectra of microdisks with and without a slit. Our findings suggest that the disks can indeed operate in whispering-gallery mode, and the slit is able to change the optical path in the microcavity without breaking lasing resonance. The slit in the microdisk can also influence the lasing mode, quality factor, and directional emission. Therefore, our study provides a feasible way to control whispering-gallery mode lasing properties.

## Introduction

Recently, the potential use of GaN as an optoelectronic device, especially in ultraviolet (UV) lasers applications, has been attracting increasingly more attention. According to the type of microcavity structure, laser devices can be classified into a few categories: random^1,2^, Fabry-Pérot (FP)^3–5^, and whispering-gallery mode (WGM) lasers^6–8^. The smooth surfaces of a WGM laser’s microcavity allow for total internal reflection with minimal optical loss, thus producing a high quality factor (Q) and a lower laser threshold than other types of structures. WGM lasing from microcavity has important potential applications in single-particle label-free sensing microdisplays used for imaging and scanning^9,10^ and in the integration of electro-optical devices onto a single wafer^11–14^. Researchers have been trying to develop a variety of microcavity structures with favorable lasing quality, mode, and directional emission, which are fundamentally important properties for the integration of optoelectronic devices. Directional radiation in lasers has been shown to be achieved by fabrication of microdisks with sharp corners or by deformation of the laser’s microcavity^15–17^. On the other hand, to achieve single-mode lasing, there are several available options: gear-shaped microdisks, distributed feedback (DFB) microstructures^18,19^, coupled cavities using the Vernier effect^20–22^, and rotationally-symmetric structures using parity-time symmetry breaking^23,24^. However, controlling both the lasing mode and directional emission in a laser device remains a challenge in the field of laser research.

In this paper, a floating GaN microdisk with a slit is proposed to control the lasing mode, Q factor, and directional emission of the lasing device by changing the optical path in the microcavity. Characteristics such as the laser threshold, mode number, Q factor, resonance mechanism, and especially the relationship between the WGM laser mode and the vertical gap in the GaN microdisk were analyzed experimentally and theoretically. In addition, we corroborate the experimental lasing spectra with the simulated light fields and emission directions to compare the GaN microdisks without or with the slit. Our study finds that the slit in the disk affects the WGM lasing mode, emission direction, Q factor, and lasing threshold. Hence, this particular structure of a microdisk laser provides a feasible way to control the WGM lasing properties.

## Results and Discussion

**Figure 1:**
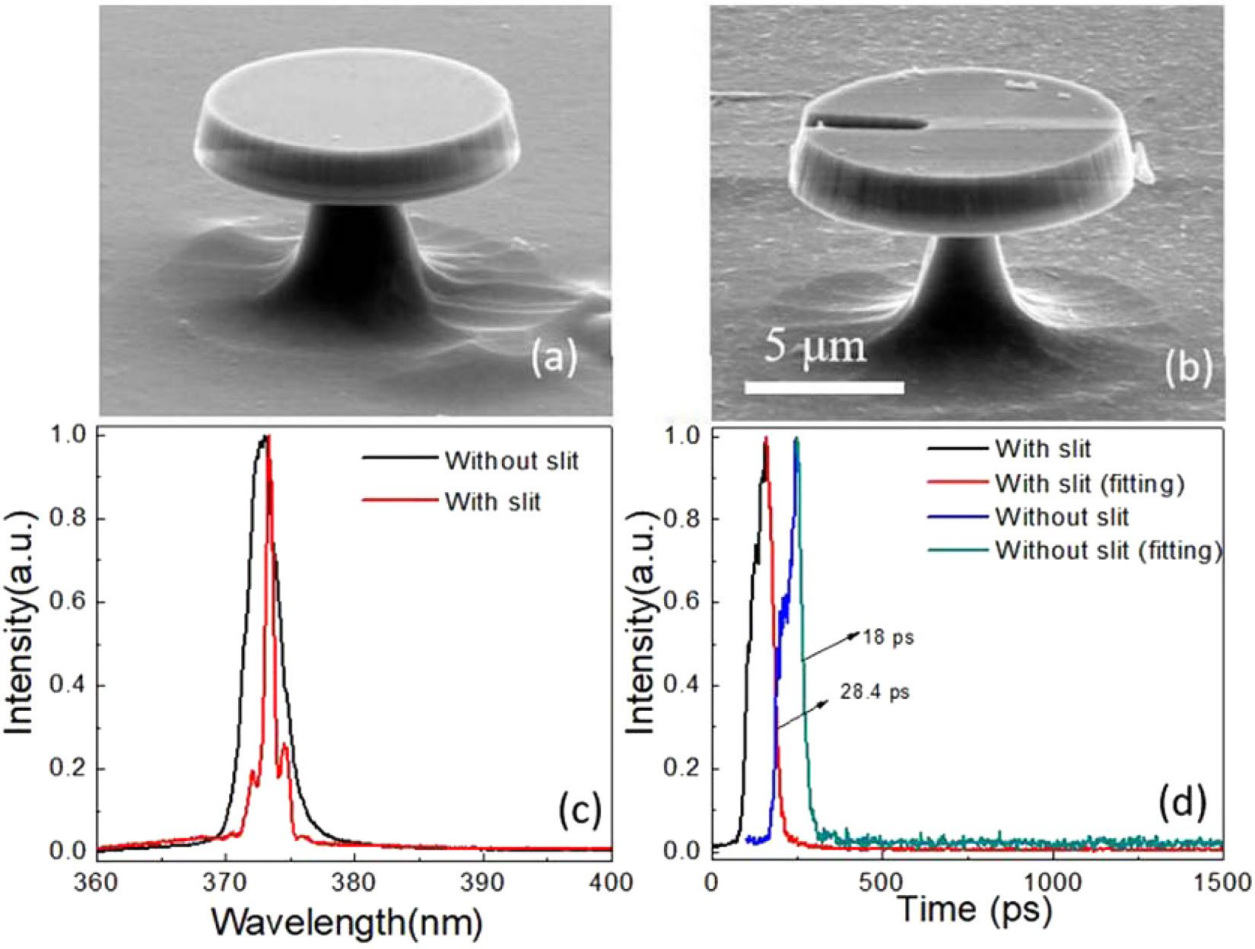
Shows the SEM images of the floating GaN microdisks, (**a**) without and (**b**) with the slit(in same scale range). Frames (**c**,**d**) consist of the corresponding normalized PL and TRPL at an excitation power of 18.4 μW.

Figure 1 displays scanning electron microscope (SEM) images of two floating GaN microdisks, without (a) and with(b) a slit, recorded by a Hitachi SU-8010 SEM. The floating diameter of the GaN microdisks is about 10 μm, the thickness 1.7 μm, and the width of the slit is 500 nm. The slit runs through the cavity along the z-axis but does not reach the central point of the microdisk. The top surface of the floating GaN microdisk is smooth, although the smoothness of the side walls is lower due to technical limitations in the etching process. The air gap between the floating microdisks and silicon is about 6 μm; in this way, the excitation light can be effectively confined in a high refractive index contrast (GaN 2.6/air 1) floating GaN microdisk. This is beneficial for achieving a low-threshold WGM laser. To understand the optical performance of GaN microdisks with or without a slit, photoluminescence (PL) and time-resolved photoluminescence (TRPL) spectra were measured by a confocal micro-photoluminescence (µ-PL) spectroscopy system and an optically-triggered streak camera system (Optronic GmbH SC-10) under fs-laser excitation (325 nm, 1000 Hz, 100 fs). As illustrated in Fig. 1(c), at an excitation power of 18.6 μW, the GaN microdisk with slit shows spontaneous radiation while the one without the slit exhibits mode oscillation. This effect is directly related to the peak with a central wavelength of 373.6 nm in the TRPL spectra in Fig. 1(d). According to single exponential fitting, the lifetime in the microdisk with the slit is 28.4 ps while in the one without the slit it is 18 ps, indicating better gain properties in the GaN microdisk without the slit.

**Figure 2:**
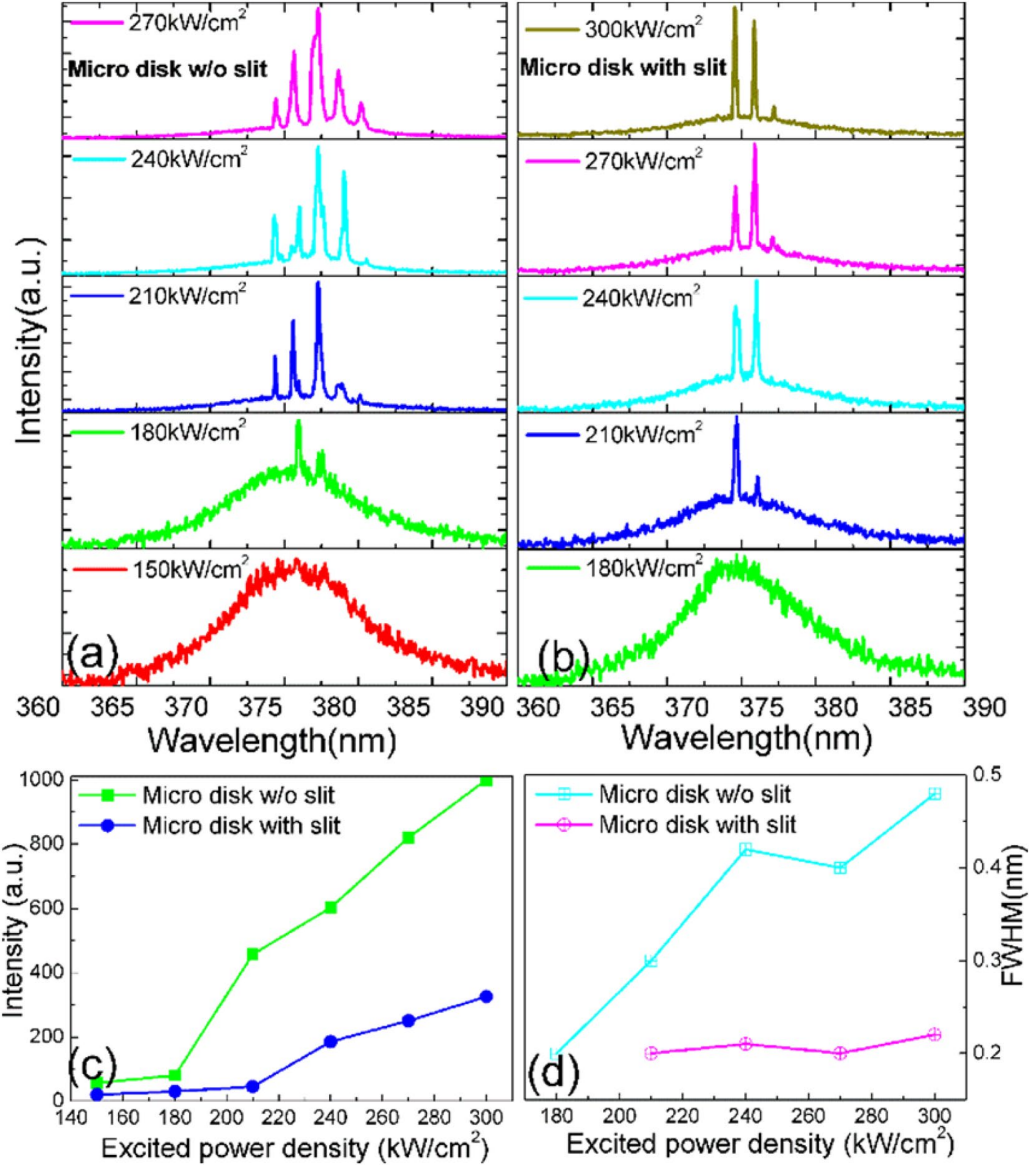
Depicts the WGM lasing spectra of the two GaN microdisks, (**a**) without and (**b**) with the slit, under different excitation pumping power densities, (**c**) the output-input relationship between the pumping power density and the lasing spectrum intensity, and (**d**) the relationship between the FWHM and the pumping power density.

The GaN microdisks with and without a slit were set as resonant cavities and their lasing performance were studied. To this purpose, a Nd:YAG pulse laser (355 nm,10 Hz, 6 ns) was employed as the excitation light source, with a diameter of the laser beam focus spot of about 10 µm. Depending on the sequence spectrum, the cavity structure can affect laser characteristics such as threshold, rate of increase in laser intensity, number of modes, and resonance position. Figure 2(a,b) show the spectra of the microdisk without and with the slit, respectively, under different pumping power densities. As shown, the floating microdisk without and with the slit show broad spectra for 150 kW/cm^2^ and 180 kW/cm^2^, respectively. This effect can be attributed to spontaneous emission in the GaN material. When the pumping power increases, for the floating microdisk without the slit, two clear lasing peaks can be observed above the excited power density of 180 kW/cm^2^, and four clear peaks above 210 kW/cm^2^. The center of the strongest laser peak is at 377.30 nm. We define the quality factor as Q = λ/Δλ, where λ and Δλ represent the center wavelength and full width at half maximum (FWHM) of the laser peak, respectively. Therefore, at an excitation power density of 210 kW/cm^2^, the Q factor for the strongest peak is about 1300. As for the floating GaN microdisk with a slit, two clear laser modes can be observed, which disappear at excited power densities above 240 kW/cm^2^. The center position of the strongest lasing peak is 376.03 nm and the FWHM is 0.2 nm. At an excitation power of 240 kW/cm^2^, the Q factor of the highest peak is about 1800. Considering the Q factors and cavity structure, the data in Fig. 2 suggests that the microdisks are operating in WGM. As the pumped power density increases, the output lasing intensity of the two types of microdisks becomes stronger, and the number of lasing peaks increases, whereas the position of the strongest resonant peak for each microdisk remains nearly the same. A small red shift of the cavity mode can be observed if we compare the lasing spectrum of the GaN microdisk with and without a slit. The free spectral range (FSR) of the cavity without the slit is about 1.22 nm and for the one with the slit 1.33 nm. For a WGM cavity, the FSR is a function of the diameter D, resonant wavelength λ, wavelength-dependent refractive index, and of the dispersion relation of the refractive index. According to Eq. 1, the increase of the FSR is caused by the slit and can be explained by the decrease in the diameter of the cavity:

1 $${\rm{FSR}}=\frac{{{\rm{\lambda }}}^{2}}{{\rm{\pi }}{\rm{D}}({\rm{n}}-{\rm{\lambda }}\frac{{\rm{dn}}}{{\rm{d}}{\rm{\lambda }}})}$$

Figure 2(c) illustrates the relationship between the laser emission intensity and the excitation power density of a floating microdisk without or with a slit. The lasing thresholds are approximately 180 kW/cm^2^ (without slit) and 210 kW/cm^2^ (with slit). When the excitation power density exceeds these lasing thresholds, the lasing emission intensities significantly increase as the excitation power density changes. This indicates that the emission spectra of the microdisks switch from spontaneous emission to stimulated radiation. The increasing rate of stimulated emission intensity of the microdisk with the slit is smaller than that of the microdisk without the slit. Moreover, the two types of microdisks exhibit different emission intensities at the same excitation power density. The plots of the threshold and intensity increase rate also show that the optical loss in the microdisk with a slit is greater than that of the microdisk without the slit. The number of lasing peaks exhibited by the two types of microdisks is also different, as shown in Fig. 2(a,b). For example, when the excitation power density is 270 kW/cm^2^, the number of laser modes in the microdisk without and with slit is 5 and 2, respectively. We note that the number of lasing modes of the microdisk with slit is always smaller than that of the microdisk without the slit.

Figure 2(d) presents the relationship between the fitted FWHM of the main lasing peak and the pumping power density. The two types of microdisks display different FWHM at the same excitation power density. The FWHM of the floating GaN microdisk with a slit is narrower than the FWHM without a slit. At the excitation power densities of 210 kW/cm^2^ and 240 kW/cm^2^, the laser Q factor of the floating GaN microdisk without and with slit is 1300 and 1800, respectively. This suggests that the slit structure can influence the lasing mode and Q factor through the following process: the slit of the microdisk generates an air gap, which changes the optical path in the microcavity and the lasing mode without damaging the lasing resonant loop. In addition, the slit width can reselect the lasing mode of the microdisk. An unusual result in Fig. 2(a,b) is that the Q factor is larger for the GaN disk with the slit. This cannot be explained by gain and loss competition, since the TRPL result in Fig. 1(d) indicates poor gain properties for the GaN disk with slit. It could be possible that the electron beam lithography (EBL) or the reactive ion etching process caused some degree of surface passivation to the slit, which in turn could have increased the Q factor of the cavity.

**Figure 3:**
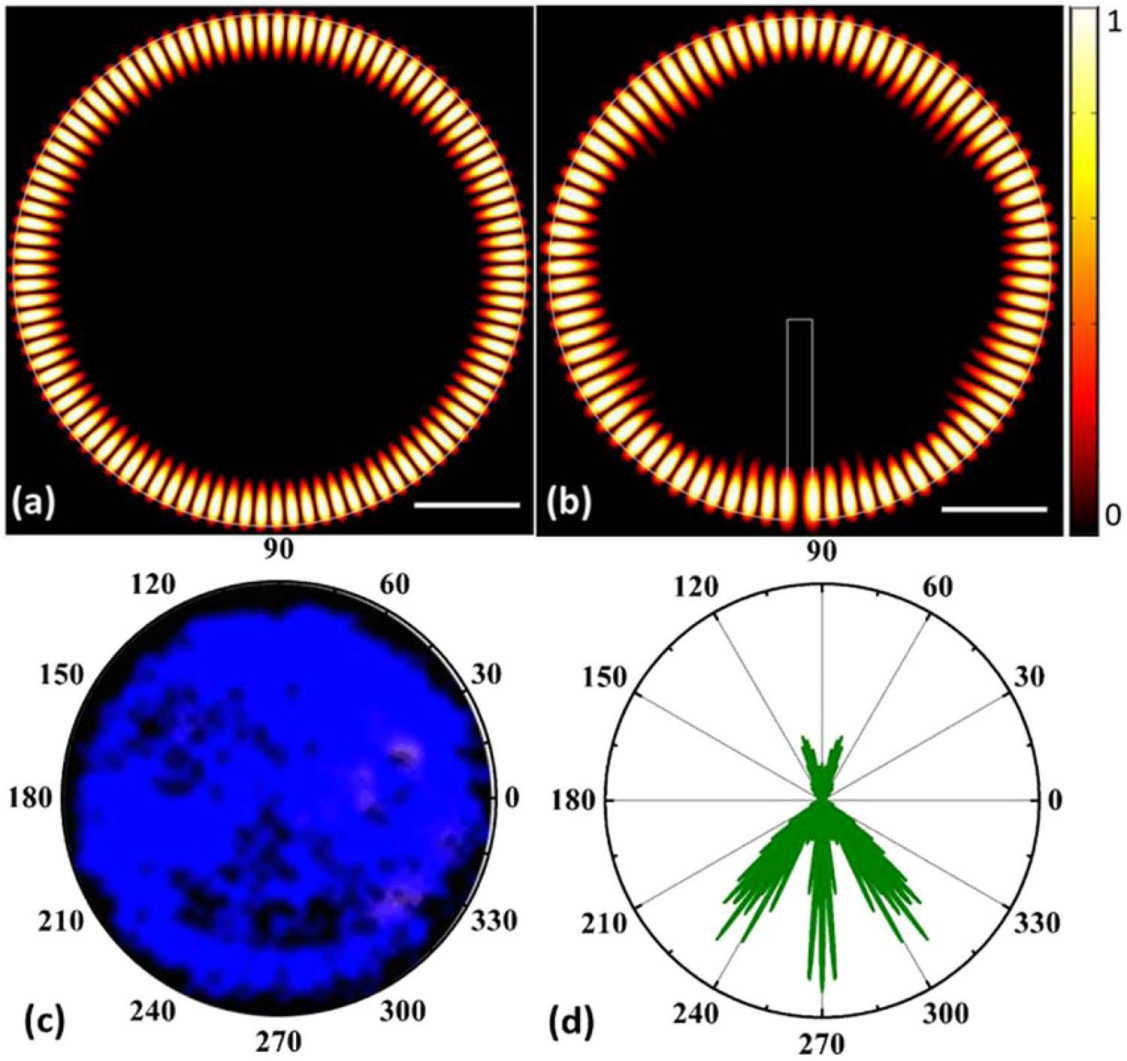
Results of the COMSOL simulation of the TE modes in the GaN microdisk without and with the vertical slit: (**a**) near-field emission pattern of the microdisk without and (**b**) with the slit, (**c**) optical image of the GaN disk with slit at 355 nm lasing excitaion, and (**d**) far-field emission pattern of the microdisk with slit.

The transverse electric field (TE) profile of the microdisk was analyzed using the commercial two-dimensional simulation software COMSOL Multiphysics^25^ to further explore the WGM laser characteristics of the two types of floating GaN microdisks. GaN is a birefringent crystal material with a hexagonal wurtzite structure. Therefore, the TM mode of the GaN microdisk in the UV range is very weak and difficult to detect compared to the TE mode. Thus, in this study, we only analyzed the TE polarized mode of the GaN microdisks. The simulated cross-sectional diameters for both types of microdisks were set to 10 μm, and the width of the slit to about 500 nm. The refractive index of GaN was taken as 2.6 at a wavelength of 375 nm. We calculated that the resonant wavelength of GaN without the slit is 377.3 nm and with the slit 376.03 nm. Figure 3(a,b) present the light field distributions of the two types of microdisks obtained from the COMSOL simulations. The resulting TE fields are circle-field distributions, which suggest that both disks operate in the WGM regime. The mode interval of the microdisk without the slit is less than that of the microdisk with the slit, which suggests that the former has a larger number of WGMs than the latter. This is consistent with the numbers of peaks in the spectra in Fig. 2(a,b). Thus, the difference in the mode intervals shows that the air gap can influence the WGM. Furthermore, the slit of the microdisk can also regulate the directional radiation of WGM lasing. The microdisk with the slit emits three-directional radiation, as shown in Fig. 3(d), which may be beneficial to its integration with other optoelectronic devices. This phenomenon can also be confirmed by the optical image of GaN with slit under a 355 nm laser excitation, as shown in Fig. 3(c). One can observe that the GaN disk with slit can also trap light in the cavity. Due to the existence of slit, light will escape through the slit.

The GaN refractive index for the TE polarization mode can be calculated using Sellmeier’s dispersion equation^26^:

2 $${n}^{2}=3.60+\frac{1.75{\lambda }^{2}}{{\lambda }^{2}-{0.256}^{2}}+\frac{4.1{\lambda }^{2}}{{\lambda }^{2}-{17.86}^{2}}$$

**Figure 4:**
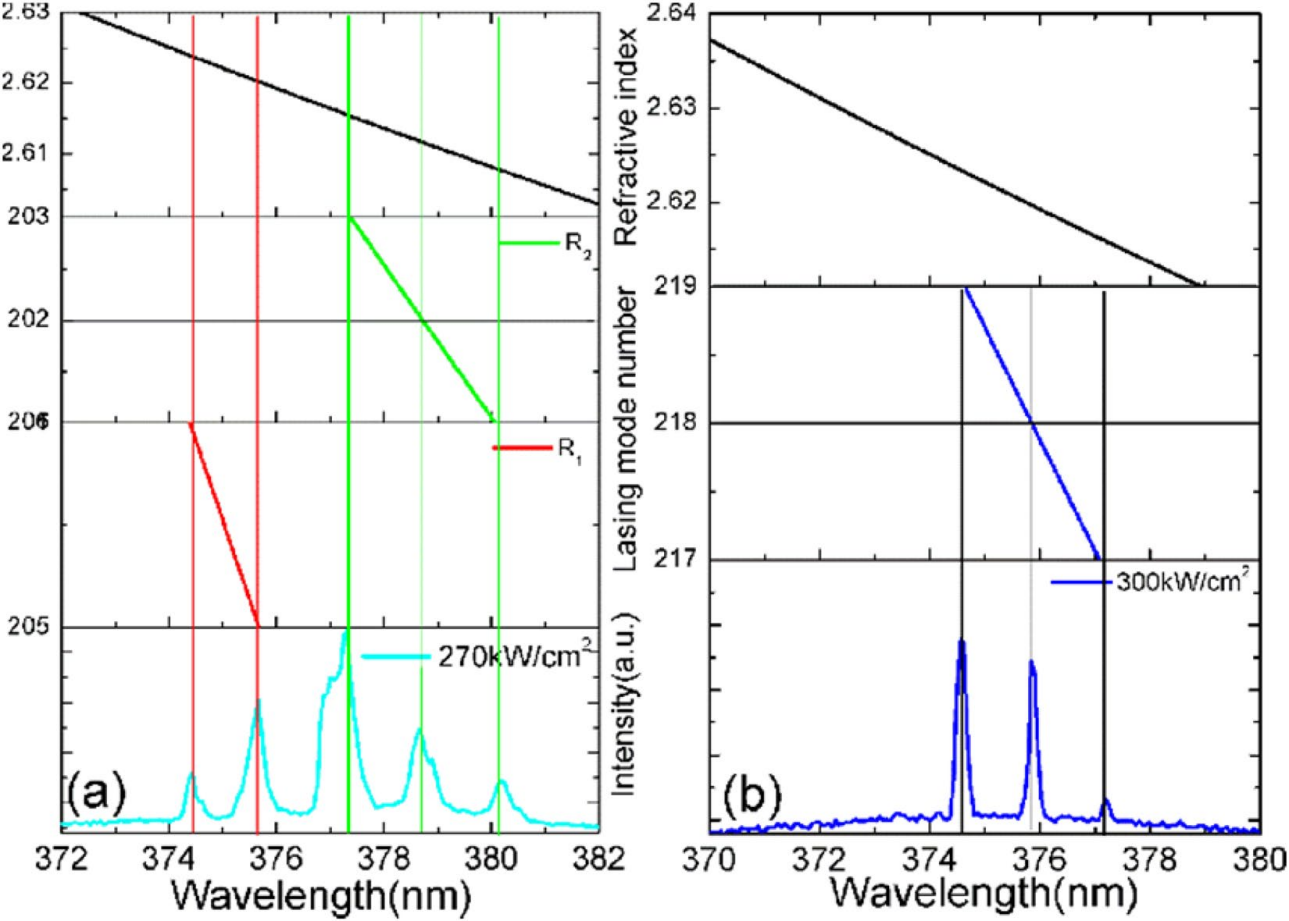
Lasing mode number of floating GaN microdisks (**a**) without and (**b**) with the slit.

The refractive index of GaN at wavelength λ is represented by n. According to the simulation results of the light field distribution, the lasing spectra from the floating GaN microdisks correspond to WGM lasing, which obeys the following mode equation^14,15^:

3 $${\rm{N}}=\frac{{\rm{\pi }}{\rm{Dn}}}{{\rm{\lambda }}}$$

where D represents the diameter of the microdisk and n is the refractive index of GaN. There are two WGMs in the lasing spectra. Accodeing to the spectra in Fig. 3 and Eq. 3, the possible diameters of the floating GaN microdisk without the slit are D_1_ = 9.35 µm and D_2_ = 9.32 µm and of the disk with slit is D_3_ = 9.95 µm. Figure 4(a) shows the dispersion relation N-λ for the WGM at the pump power density of 270 kW/cm^2^. The N-λ curve describes the relationship between the number N of lasing emission modes and the wavelength λ. The intersection point of any vertical line with the lasing spectra and the N-λ curve is an integer, which is exactly the number of lasing modes for the corresponding peak. This confirms that we obtain WGM lasing in our device. The dispersion diagram also illustrates the refractive index of GaN at the different peaks. We index the lasing modes of the GaN microdisk without the slit as follows: 205, 206 for diameter D_1_ and 201, 202, and 203 for D_2_, as shown in Fig. 4(a). Similarly, Fig. 4(b) presents the dispersion relation N-λ for the WGMs in the microdisk with the slit and at a pumping power density of 300 kW/cm^2^. The lasing modes are indexed as follows: 205 and 206 for the GaN microdisk without slit and diameter D_1_ and 217, 218, and 219 for the microdisk with slit and diameter D, as shown in Fig. 4(b). The experimentally-determined resonant wavelengths are in good agreement with our simulations of the light field distributions.

To summarize, we synthesized two types of floating GaN microdisks, without and with a slit, by GaN dry etching and HNF solution isotropic wet etching, respectively. The silicon substrates were etched by photolithography. Subsequently, UV WGM lasing was obtained by optical pumping. The present study finds that the floating GaN microdisk with a slit is able to influence the lasing mode, Q factor, and directional emission, perhaps due to the fact that the slit structure changes the optical path in the microdisk. Furthermore, we investigated the resonance mechanism and the TE light field distribution of the two types of floating GaN microdisks using COMSOL simulations. Hence, our work contributes to the fields of light-matter interactions and integrated photovoltaic systems by providing a feasible way to control the whispering-gallery mode lasing properties in a floating GaN microdisk.

### Experimental details

**Figure 5:**
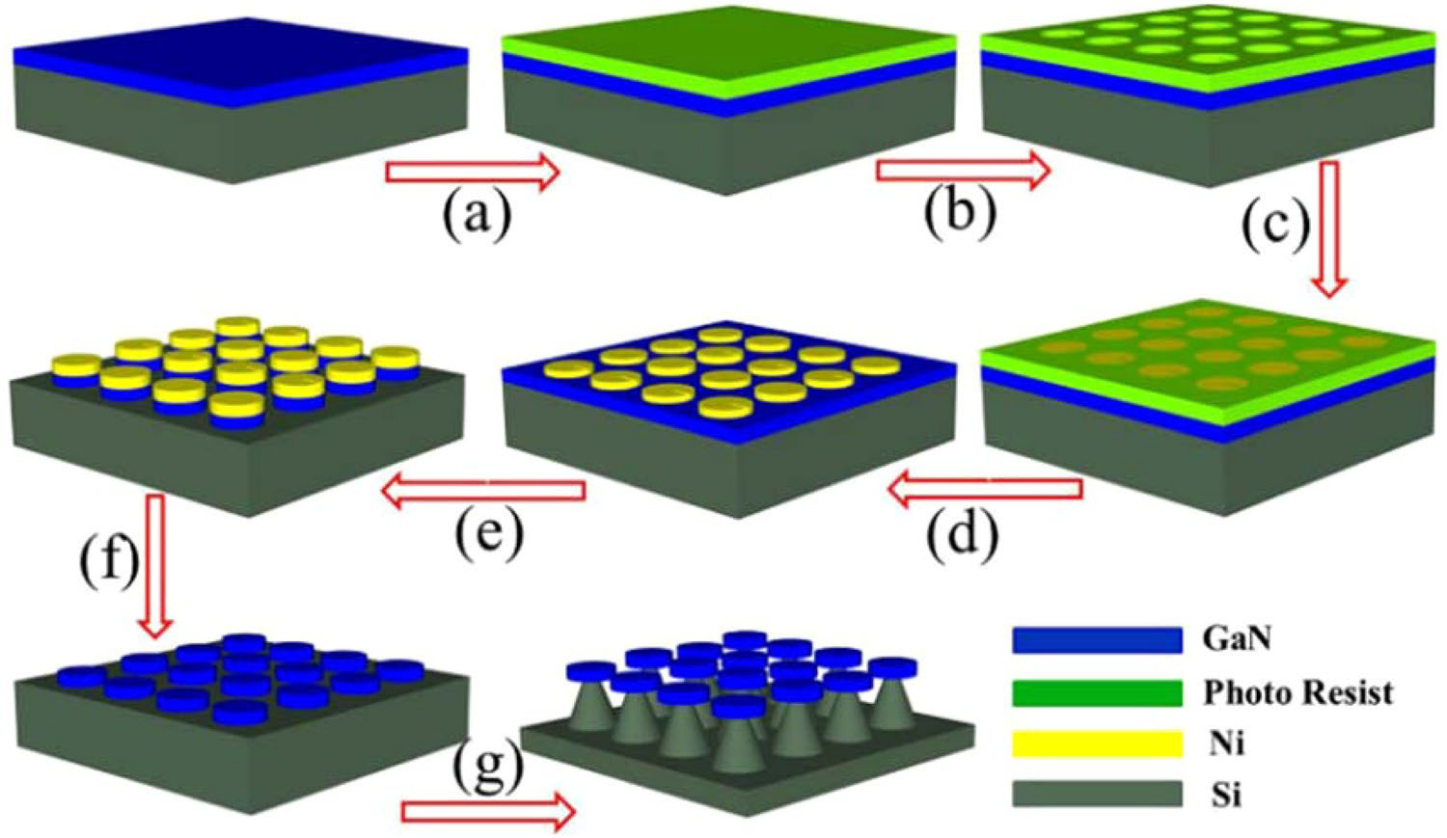
The schematic diagram of the manufacturing process of a floating GaN microdisk.

The floating GaN microdisks were fabricated on a commercial GaN-on-silicon wafer, with the following thicknesses of the component layers: 1.7 μm for GaN and 1500 μm for the silicon substrate. The manufacturing process of the floating GaN microdisk is as shown in Fig. 5. First, a pattern was defined on a wafer by electron beam lithography. Subsequently, a 230 nm hard mask of metallic nickel was deposited by magnetron sputtering (steps a, b, and c). After the residual photoresist was removed (step d), a 1.7 μm etch was performed from the top (step e), and the wafer was etched to the silicon layer. The specific parameters of the reactive ion etching (ICP) etching process were as follows. The flow rates were 30 sccm for argon, 20 sccm for chlorine, and 20 sccm for boron trichloride. The applied power was set to 500 W and the pressure to 60 mTorr for 7 minutes; the etching rate of GaN was about 300 nm/minute. After removing the residual nickel film with a dilute nitric acid solution (step f), the GaN microdisk was undercut using an HNF (HF:HNO_3_ = 1:9) isotropic wet-etching solution and a large air gap (5 µm) was created under the GaN microdisk. A small silicon pillar under the disk was retained for mechanical support. Along the vertical direction of the GaN microdisk, the optical confinement capability of the fabricated device was improved, and the optical loss due to the removal of the silicon substrate was reduced. Finally, the GaN microdisk was suspended in air by the silicon pillar support (step g). The GaN microdisk with the slit was fabricated by following the exact same steps, with the only difference that a pattern with a slit was used in the first step.

## References

[1] Li X, Wang Y, Sun H, Zeng H (2017). Amino-Mediated Anchoring Perovskite Quantum Dots for Stable and Low-Threshold Random Lasing. Adv. Mater..

[2] Chang T-C (2017). High-temperature operation of GaN-based vertical-cavity surface-emitting lasers. Appl. Phys. Express.

[3] Li J (2015). Plasmon coupled Fabry-Perot lasing enhancement in graphene/ZnO hybrid microcavity. Sci. Rep..

[4] Baek H, Hyun JK, Chung K, Oh H, Yi G-C (2014). Selective excitation of Fabry-Perot or whispering-gallery mode-type lasing in GaN microrods. Appl. Phys. Lett..

[5] Wang X (2018). High-Quality In-Plane Aligned CsPbX_3_ Perovskite Nanowire Lasers with Composition-Dependent Strong Exciton - Photon Coupling. Acs. Nano..

[6] Wang LC (2017). Optically pumped lasing with a Q-factor exceeding 6000 from wet-etched GaN micro-pyramids. Opt. Lett..

[7] Pan AL (2005). Optical waveguide through CdS nanoribbons. Small..

[8] Yang Y-D (2019). Whispering-gallery mode hexagonal micro-/nanocavity lasers Invited. Photonics Res..

[9] Knapper KA, Heylman KD, Horak EH, Goldsmith RH (2016). Chip-Scale Fabrication of High-Q All-Glass Toroidal Microresonators for Single-Particle Label-Free Imaging. Adv. Mater..

[10] Heylman KD, Knapper KA, Goldsmith RH (2014). Photothermal Microscopy of Nonluminescent Single Particles Enabled by Optical Microresonators. J. Phys. Chem. Lett..

[11] Feng M (2018). On-chip integration of GaN-based laser, modulator, and photodetector grown on Si. IEEE J. Sel. Top. Quant..

[12] Wang Y (2017). Chip-Scale Mass Manufacturable High-Q Silicon Microdisks. Adv. Mater. Technol..

[13] Li G (2015). High-Q silica microdisk optical resonators with large wedge angles on a silicon chip. Photonics Res.

[14] Stock E (2013). On-chip quantum optics with quantum dot microcavities. Adv. Mater..

[15] Zhu GY, Qin FF, Guo JY, Xu CX, Wang YJ (2017). Unidirectional ultraviolet whispering-gallery mode lasing from floating asymmetric circle GaN microdisk. Appl. Phys. Lett..

[16] Jiang X-F, Zou C-L, Wang L, Gong Q, Xiao Y-F (2016). Whispering-gallery microcavities with unidirectional laser emission. Laser Photonics Rev..

[17] Kneissl M (2004). Current-injection spiral-shaped microcavity disk laser diodes with unidirectional emission. Appl. Phys. Lett..

[18] Nozaki K, Nakagawa A, Sano D, Baba T (2003). Ultralow threshold and single-mode lasing in microgear lasers and its fusion with quasi-periodic photonic crystals. IEEE Journal of Selected Topics in Quantum Electronics.

[19] Baba MFaT (2002). Microgear laser. Applied Physics Letters.

[20] Yang C, Hu Y, Jiang X, Xiao M (2017). Analysis of a triple-cavity photonic molecule based on coupled-mode theory. Physical Review A.

[21] Shang L, Liu L, Xu L (2008). Single-frequency coupled asymmetric microcavity laser. Opt. Lett..

[22] Wu X, Li H, Liu L, Xu L (2008). Unidirectional single-frequency lasing from a ring-spiral coupled microcavity laser. Appl. Phys. Lett..

[23] Song Q (2015). The combination of directional outputs and single-mode operation in circular microdisk with broken PT symmetry. Opt. Express.

[24] Feng L, Wong ZJ, Ma RM, Wang Y, Zhang X (2014). Single-mode laser by parity-time symmetry breaking. Science.

[25] Zhu G (2018). Single-mode ultraviolet whispering-gallery mode lasing from a floating GaN microdisk. Opt. Lett..

[26] Barker AS, Ilegems M (1973). Infrared Lattice Vibrations and Free-Electron Dispersion in GaN. Physical Review B.

